# Helicobacter pylori-Negative Differentiated Intramucosal Gastric Cancer in the Antrum With a Morphological Change in Four Years: A Case Series

**DOI:** 10.7759/cureus.63148

**Published:** 2024-06-25

**Authors:** Takashi Obana, Shuuji Yamasaki, Yoshikazu Yakami

**Affiliations:** 1 Department of Gastroenterology, Kyojinkai Komatsu Hospital, Osaka, JPN

**Keywords:** gastric cancer, helicobacter pylori-negative, intestinal type, morphological change, natural course

## Abstract

This case report presents two cases of differentiated intramucosal gastric cancer in the antrum. Both patients reported no history of *Helicobacter pylori* eradication therapy, and endoscopy and diagnostic tests indicated no *H. pylori* infection. Case 1 is a female patient in her 70s. Esophagoduodenogastroscopy (EGD) detected a depressed lesion. Adenocarcinoma was suspected; thus, endoscopic submucosal dissection (ESD) was performed to resect the lesion. The histological result was well-differentiated tubular adenocarcinoma, which predominantly demonstrated an intestinal mucin phenotype. The existence of a small elevated lesion in the same location was confirmed by reviewing the previous endoscopic record 52 months earlier. Case 2 is a male patient in his 60s in whom screening EGD detected an elevated lesion. The biopsy indicated gastric adenoma, and ESD was performed. The histological diagnosis was well-to-moderately differentiated tubular adenocarcinoma with a pure gastric phenotype. These results indicate that *H. pylori*-negative differentiated gastric carcinomas in the antrum occur as small elevated lesions that may gradually progress to a depressed form during a relatively long clinical course.

## Introduction

*Helicobacter pylori *is designated as a “definite carcinogen” by the World Health Organization and is known to play a key role in the development of gastric cancer [1]. Although most gastric carcinomas occur in *H. pylori*-positive patients [2], *H. pylori*-negative cases have also been recognized [3]. Since the introduction of *H. pylori* eradication therapy, attention has gradually become focused on *H. pylori*-negative gastric neoplasms. Among them, those that originate from the antrum and demonstrate differentiated histology have been reported. These lesions sometimes exhibit an intestinal mucin phenotype [4]. The natural course or mode of progression has not been elucidated. Here, we present two cases of *H. pylori*-negative differentiated early gastric cancer in the antrum, one of which demonstrated a morphological change in four years.

## Case presentation

**Figure 1:**
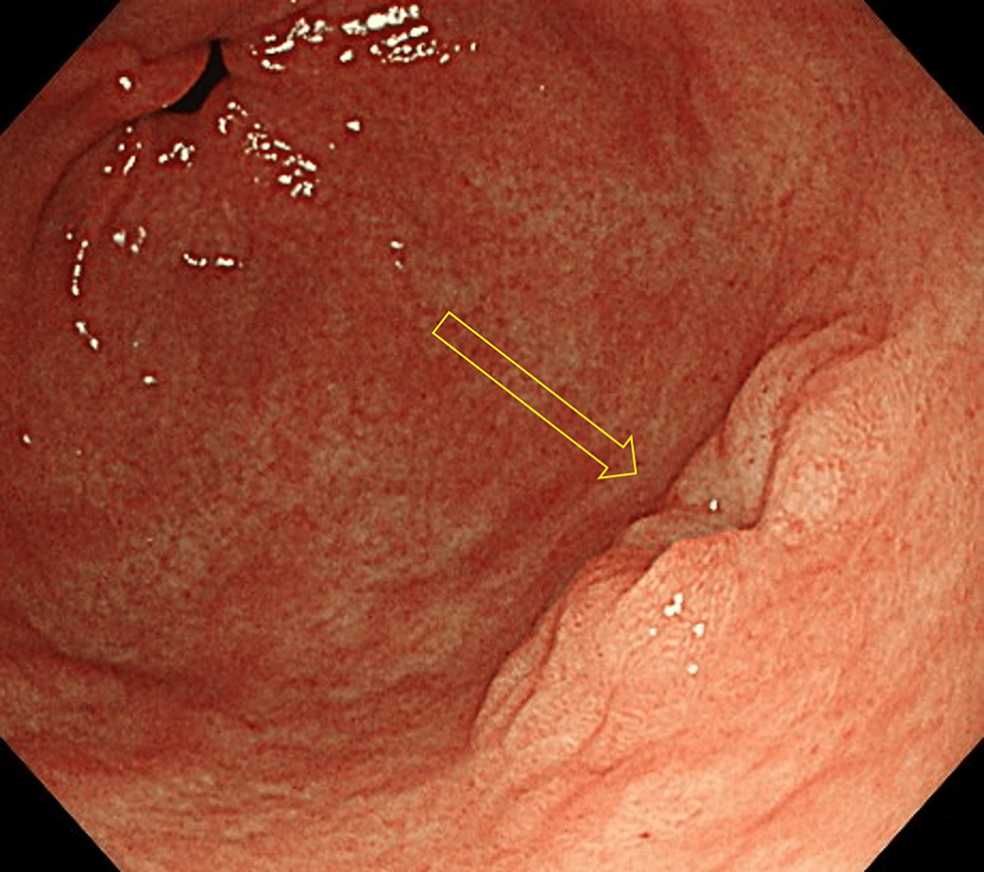
Endoscopic image of Case 1 Esophagoduodenogastroscopy (EGD) detected a depressed lesion on the greater curvature of the gastric antrum.

**Figure 2:**
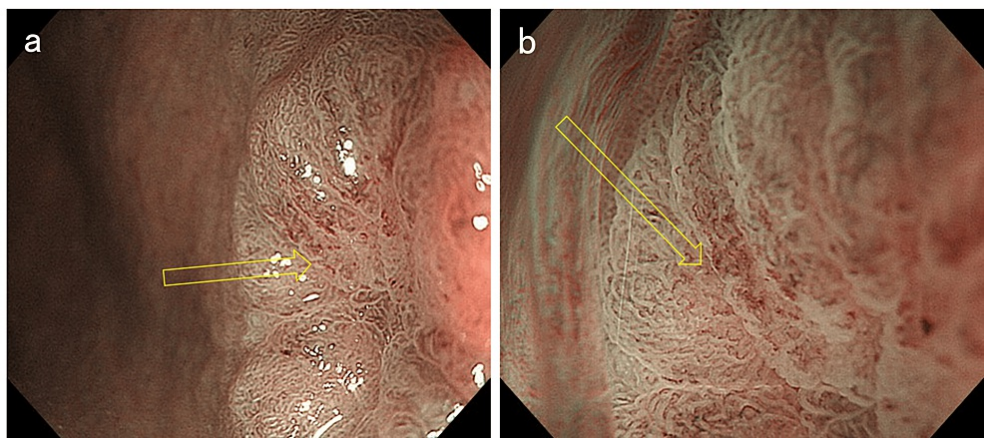
Narrow-band imaging (NBI) findings of the lesion a. Magnifying endoscopy with narrow-band imaging (ME-NBI) shows an irregular microsurface pattern in the depressed area. b. A slightly irregular microvascular pattern was also observed.

Case 1 is a female patient in her 70s who was under treatment for diabetes mellitus and reflux esophagitis. The patient was taking a proton pump inhibitor (vonoprazan fumarate) and was a current smoker of 10 cigarettes per day. Esophagoduodenogastroscopy (EGD) was performed as an endoscopic follow-up for reflux esophagitis and detected a depressed lesion on the greater curvature of the gastric antrum (Figure 1). Magnifying endoscopy with narrow-band imaging (ME-NBI) detected slightly irregular microsurface and microvascular patterns (Figure 2). The background gastric mucosa was endoscopically nonatrophic. Biopsy samples were obtained, and the existence of adenocarcinoma cells was suspected.

**Figure 3:**
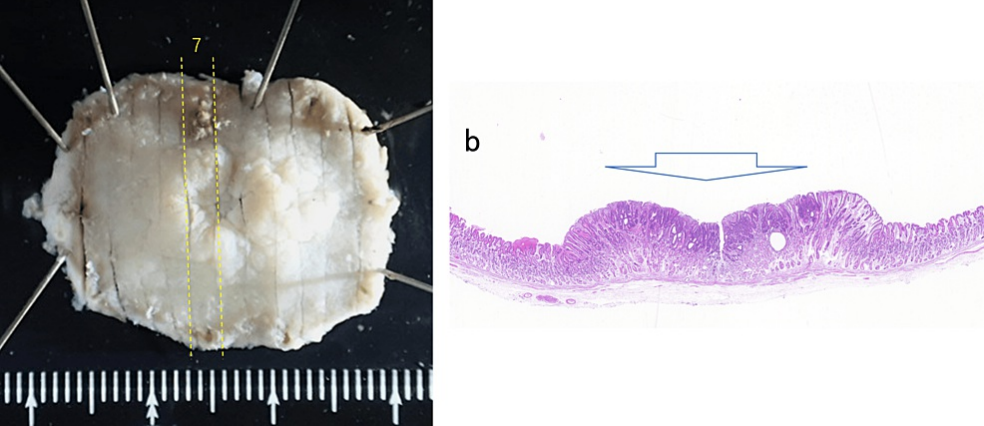
Resected specimen a. The resected specimen was cut into 12 slices. b. In slice 7, neoplastic cells were present in the denoted area (hematoxylin-eosin (HE) staining).

**Figure 4:**
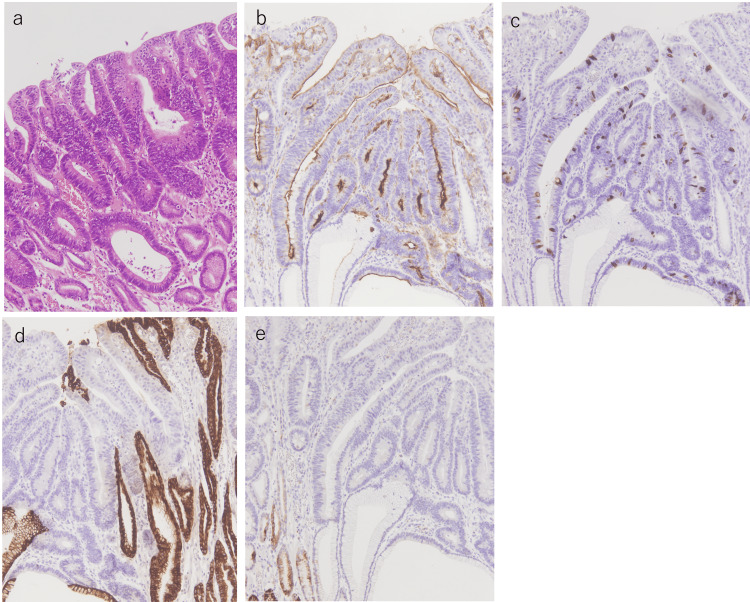
Histopathological images a. The high-power field of slice 7 reveals the existence of well-differentiated tubular adenocarcinoma (hematoxylin and eosin (HE) ×100). b-e. Immunohistochemical staining exhibiting positive CD10 (b) and MUC2 (c) expression. MUC5AC was partially stained (d), and MUC6 was not stained (e),  indicating a predominantly intestinal phenotype (×100).

**Figure 5:**
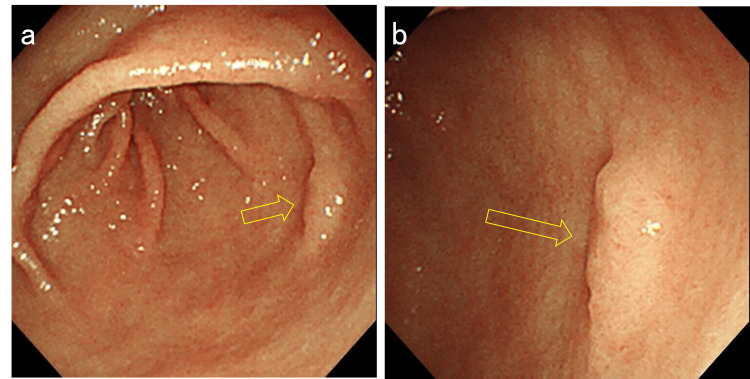
Endoscopic images of the lesion 52 months earlier a. Reviewing the previous endoscopic record 52 months earlier revealed the existence of a small elevated lesion in the same location. b. A closer view of the lesion.

Endoscopic submucosal dissection (ESD) was performed to resect the lesion. The histological result was intramucosal well-differentiated tubular adenocarcinoma without lymphovascular invasion. The tumor was 6 mm in size (Figure 3). She reported no medical history of *H. pylori* eradication therapy, and the results of two *H. pylori* diagnostic tests, namely, serum immunoglobulin G (IgG) anti-*H. pylori* antibody and fecal *H. pylori* antigen levels, were negative. Immunohistochemical staining revealed that neoplastic cells expressed CD10 and MUC2. Conversely, MUC5AC was partially stained and MUC6 was not expressed (Figure 4). These findings indicated that the lesion was differentiated gastric cancer with a predominantly intestinal mucin phenotype that developed in a patient with negative *H. pylori*. Intestinal metaplasia was not observed in the surrounding mucosa. The existence of a small elevated lesion in the same location was confirmed by reviewing the previous endoscopic record 52 months earlier (Figure 5). A morphological change from an elevated to a depressed form during this period was indicated. The patient has been followed up without any sign of recurrence for two years after treatment.

**Figure 6:**
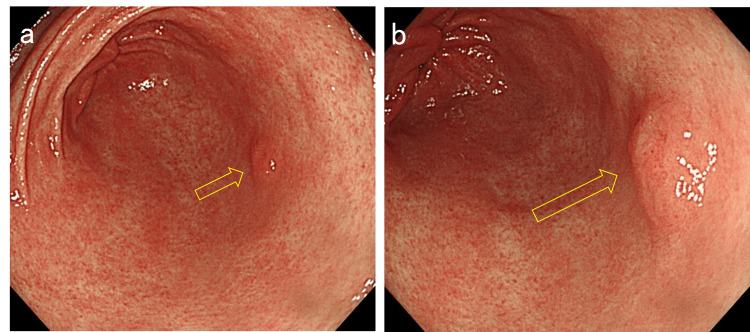
Endoscopic images of Case 2 a. Esophagoduodenogastroscopy (EGD) showing an elevated lesion in the antrum. The morphology and location of the lesion were quite similar to those in Figure 5. b.  A closer view of the lesion.

**Figure 7:**
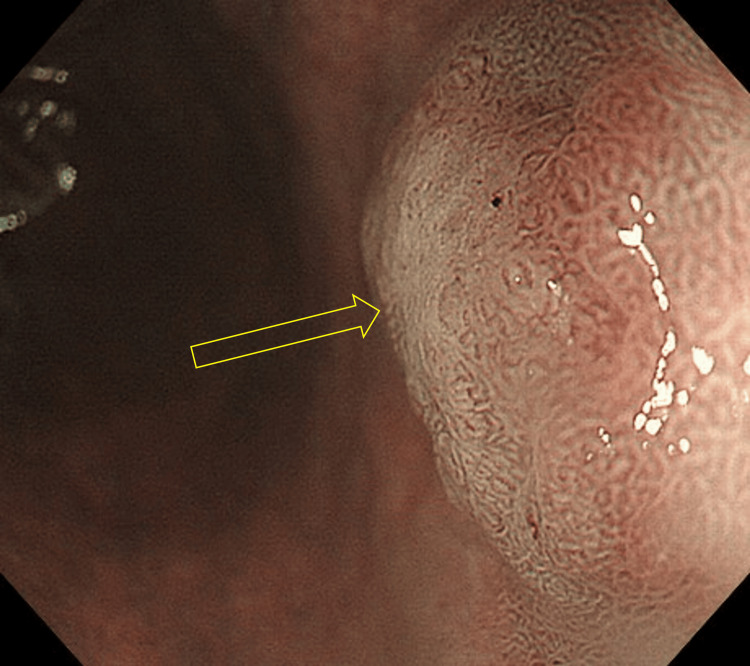
Narrow-band imaging (NBI) findings Magnifying endoscopy with narrow-band imaging (ME-NBI) shows an irregular microsurface pattern. The irregularity of the microvascular pattern was not confirmed.

**Figure 8:**
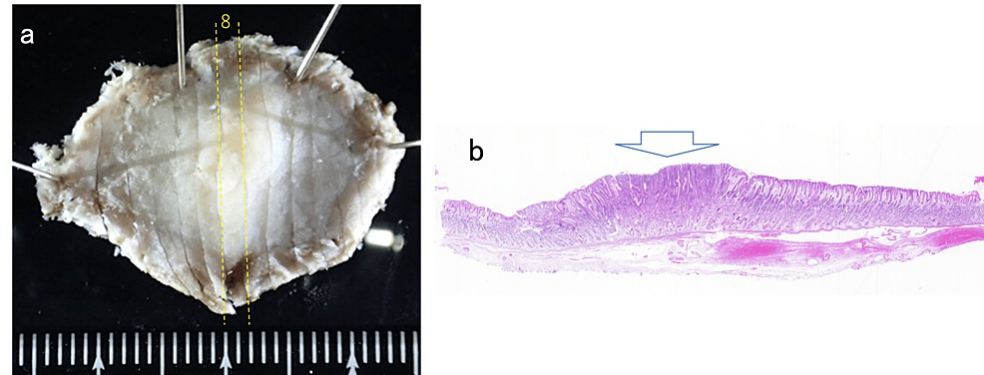
Resected specimen a. The resected specimen was cut into 14 slices. b. In slice 8, adenocarcinoma cells were present in the denoted area (hematoxylin and eosin (HE) staining).

**Figure 9:**
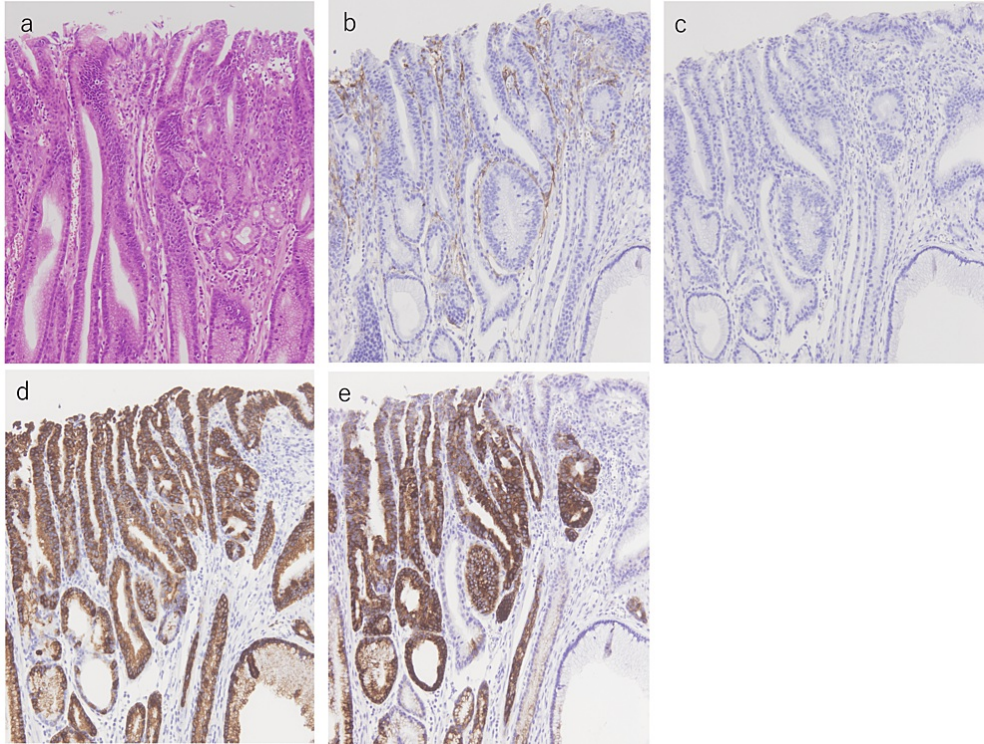
Histopathological images a. The high-power field of slice 8 reveals a well-to-moderately differentiated tubular adenocarcinoma (hematoxylin and eosin (HE) ×100). b-e. Immunohistochemical staining exhibiting negative CD10 (b) and MUC2 (c) expression. By contrast, MUC5AC (d) and MUC6 (e) were positively stained, indicating a pure gastric phenotype (×100).

Case 2 is a male patient in his 60s who has been on medication for hypertension and dyslipidemia. The patient used to smoke but quit several years ago. Screening EGD detected an elevated lesion on the greater curvature of the antrum in a nonatrophic stomach. The lesion morphology and location were quite similar to those of Case 1 at 52 months earlier (Figure 6). The microsurface pattern of the lesion was slightly irregular by ME-NBI, but differentiating it from a raised erosion was difficult (Figure 7). The biopsy indicated gastric adenoma, but ESD was performed on the suspicion of adenocarcinoma. The pathological diagnosis was well-to-moderately differentiated tubular adenocarcinoma confined to the mucosa. The tumor was 3 mm in size (Figure 8). Lymphovascular invasions were not detected, and intestinal metaplasia was absent in the surrounding mucosa. Only MUC5AC and MUC6 were positively stained immunohistochemically (Figure 9). The patient reported no medical history of *H. pylori *eradication therapy, and the results of two *H. pylori* diagnostic tests, namely, serum IgG anti-*H. pylori* antibody level and 13C-labeled urea breath tests, were negative. Given these findings, the lesion was finally diagnosed as differentiated gastric cancer with a pure gastric mucin phenotype, which arose in a patient with negative *H. pylori*. After treatment, he has been under follow-up without recurrence for five months.

## Discussion

The prevalence of *H. pylori*-negative gastric cancer is estimated to be approximately 1%, which differs according to definitions [3]. Several subtypes of *H. pylori*-negative gastric cancer have been reported, such as signet-ring cell, fundic-gland, or foveolar-type carcinoma [5,6]. Recently, differentiated gastric carcinoma in the antrum that sometimes expresses an intestinal mucin phenotype has been recognized as another entity [4,7]. This type of lesion shows flat-elevated or depressed morphology, mimicking varioliform gastritis. Shibagaki et al. confirmed multiple growths in 30%, NBI-ME showing a near-regular microsurface/microvascular pattern in 50%, and sporadic intestinal metaplasia was observed histologically in 40% of the cases [4]. The depth of the reported cases in Japan has been intramucosal, except for one patient [7,8]. The pathogenesis has not been elucidated, although several reports hypothesize that bile acid reflux may play a role in carcinogenesis [4,9,10]. The natural course of these lesions remains to be investigated.

The present two cases showed a similar elevated appearance that was difficult to differentiate from a raised erosion or reactive hyperplasia at the time of the first endoscopy. This type of mucosal change could be overlooked without biopsies, especially in the low-risk cohort of patients with gastric carcinoma, namely, *H. pylori *negative. In Case 1, the tumor progressed to a depressed form after 52 months. The clinical course indicates the possibility that *H. pylori*-negative differentiated gastric cancer in the antrum may originate as an elevated lesion and then morphologically progress to a depressed one during a relatively long period. The depth of invasion was still intramucosal, which also implies its slow progression. Similarly, Takita reported that none of their cases demonstrated rapid growth in four to 11 years [7]. Mucin phenotypic shifting from gastric to mixed or intestinal type may also occur during the progression. In clinical practice, biopsy should be considered when a single elevated lesion is detected in the antrum of patients with endoscopically negative *H. pylori*, even if ME-NBI did not reveal convincing findings. If the lesions are multiple, performing biopsies from all sites would not be feasible.

Fortunately, regular follow-up by EGD is considered acceptable in light of the relatively slow progression of these lesions. The influence of bile acid reflux was not verified in these particular cases because intestinal metaplasia was not detected in either case. The impact of other carcinogens, such as cigarettes, should also be evaluated by the further accumulation of cases. In the present cases, the *H. pylori*-negative status was judged based on the following findings: (1) no gastric mucosal atrophy evaluated by endoscopy, (2) negative results of two tests for *H. pylori* infection, and (3) no medical history of *H. pylori *eradication therapy. Thus far, the diagnostic rule of *H. pylori* negativity has not been established. Yamamoto proposed diagnostic criteria that include pathological findings (the updated Sydney System) and serum pepsinogen test [3]. In Case 1, three biopsy specimens were obtained from the greater curvature of the antrum, incisura angularis, and greater curvature of the corpus, and then a single pathologist evaluated them using the updated Sydney System. The gradings were rated mild for atrophy and mononuclear cells, and normal (none) for neutrophils, *H. pylori,* and intestinal metaplasia. However, histological grading is judged by the visual analog scale in the updated Sydney System, and the interobserver agreement, especially for the degree of atrophy, is reported to be relatively low [11,12]. In view of the obscure boundary between “normal” and “mild," we consider that the grading results do not deny the *H. pylori*-negative status in Case 1. The patient in Case 2 was diagnosed as *H. pylori*-negative by the minimum criteria proposed by Yamamoto because the result of the serum pepsinogen test was also negative.

## Conclusions

*H. pylori*-negative gastric carcinomas are relatively rare, and early diagnosis is sometimes difficult. Although this category includes several subtypes, the occurrence and progression of them have not been clarified. The present cases are clinically important because they indicate that *H. pylori*-negative differentiated gastric cancer in the antrum may originate as a small elevated lesion and then gradually progress to a depressed form over a relatively long time. Further accumulation of cases is necessary to elucidate the natural course of this disease entity.
